# Memantine-induced speech problems in two patients with autistic disorder

**DOI:** 10.1186/2008-2231-21-54

**Published:** 2013-07-02

**Authors:** Javad Alaghband-Rad, Naemeh Nikvarz, Mehdi Tehrani-Doost, Padideh Ghaeli

**Affiliations:** 1 Roozbeh Hospital, Department of Psychiatry, Faculty of Medicine, Tehran University of Medical Sciences (TUMS), Tehran, Iran; 2 Department of Pharmacotherapy, Faculty of Pharmacy, Kerman Medical University (KMU), Kerman, Iran; 3 Research Center for Rational Use of Drugs and Department of Clinical Pharmacy, Faculty of Pharmacy, Tehran University of Medical Sciences (TUMS), Tehran, Iran

**Keywords:** Stuttering, Autism spectrum disorder, Memantine

## Abstract

Stuttering is a complex speech disorder. There are two forms of stuttering: developmental stuttering and acquired stuttering. Developmental stuttering is a disorder of early childhood but acquired stuttering can develop at any age. Some medications can induce or deteriorate stuttering as an adverse effect. There are several reports of stuttering due to psychotropic drugs. Memantine, a glutamate antagonist used in the treatment of Alzheimer’s disease, has also been studied for the treatment of autism spectrum disorders. This report presents deterioration of stuttering and speech problem in two children with autistic disorder who were receiving memantine. Based on our knowledge, this is the first time these adverse drug reactions have been attributed to memantine. In conclusion clinicians should consider that speech problems including stuttering may be due to the consumption of memantine, especially, in children may be a side effect of memantine especially in children.

## Background

Stuttering is a neurodevelopmental disorder which interferes with academic and/ or occupational achievement as well as social communication.

The International Classification of Diseases version 10 (ICD-10) categorized stuttering as a behavioral and emotional disorder, and coded as F98.5 with this depiction: “Speech that is characterized by frequent repetition or prolongation of sounds or syllables or words, or by frequent hesitations or pauses that disrupt the rhythmic flow of speech. It should be classified as a disorder only if its severity is such as to markedly disturb the fluency of speech”.

Diagnostic and Statistical Manual of Mental Disorders, fourth Edition, Text Revision (DSM-IV-TR) described stuttering as frequent occurrences of one or more of the following: sound and syllable repetitions, sound prolongations, interjections, broken words, audible or silent blocking, circumlocutions, words produced with an excess of physical tension, and monosyllabic whole word repetitions.

Developmental stuttering, the most common type of stuttering, is a disorder of early childhood with mean age of onset of approximately 33 months. Yairiand Ambrose, in a recent review article approximated the life-time prevalence of stuttering as 0.75% and its life time incidence has been reported to be between 5 to 8%. This study also reported that the prevalence of this disorder is a little higher among men at the onset of this disorder that what is seen in females. “The smaller polarity of affected males versus females near the time of onset as compared with the polarity at more advanced ages suggests that recovery from stuttering is considerably more frequent in girls than in boys” [1]. Recovery from developmental stuttering without any intervention occurs in about 75% of persons [2]. Acquired stuttering which can occur at any age is resulted from secondary causes such as drugs, head trauma, stroke, and brain tumors [2-4]. Most published reports of drug induced stuttering have noted psychotropics such as bupropion [5], clozapine [6,7], topiramate [8], lithium [4], tricyclic antidepressants [5,9], phenothiazines [5,9,10], selective serotonin reuptake inhibitors (sertraline and fluoxetine) [5,9], risperidone [11], olanzapine [7], stimulants [9,10,12] as responsible agents.

Autistic disorder is a subtype of pervasive developmental disorders (PDDs). The core symptoms of autism include impairments in social interactions, verbal and nonverbal communication skills and stereotyped actions and tendencies [13]. An autistic patient that is able to speak may have some language or speech disorders. Many PDDs patients have structural language disturbances or Functional deficits [14]. Stuttering is one of the speech disorders that may occur in PDDs patients [15,16]. Memantine, an antagonist of N-Methyl-D-Aspartate (NMDA) receptors of glutamate [17], was approved for the treatment of moderate to severe Alzheimer’s disease. It has been used in some patients with PDDs [18-21]. Our report includes two cases with speech difficulty and deterioration of stuttering caused by memantine that was prescribed for the management of symptoms of autistic disorder.

## Case presentation

### Case1

A 9-year-old boy, with a 5 years history of autistic disorder, was referred to one of the author’s child and adolescent psychiatric clinic (J.A.) for the management of his condition. His parents report his developmental stuttering as sound repetition and sound prolongation on first and middle vowels since age 4. At the time the child psychiatrist prescribed risperidone (an atypical antipsychotic); however, the parents refused giving this medication to their son because of their general worries about the side effects of drugs. Over time, the parents came to conclusion that they need to seek professional psychiatric help for their child’s behavioral and educational difficulties. After a thorough assessment of the patient, memantine 5 mg per day was started and increased to 7.5 mg per day after 7 days. The dose was increased to 10 mg per day once again after 1 week. At the beginning of the third week, he encountered deterioration of stuttering and difficulty for starting to speak. His parents explained that the child could only start to speak after a deep and audible breath. Otherwise, he was not able to talk. This problem was only noted at the beginning of the patient’s speech and vanished after a while. No other new stressful or deteriorating condition was thought to aggravate his speech difficulty. Since memantine was the only drug he was consuming, it was decided to reduce the dose of memantine to 7.5 mg per day. Several days after this dose reduction, the patient did not need to take such a deep, audible breath before speaking. However, the deteriorating symptoms of stuttering caused so much distress for the patient and his family that the treating psychiatrist decided to taper down and finally to discontinue memantine. Three weeks after discontinuation of memantine, aggravated stuttering was reduced to its baseline. Then, risperidone was prescribed for the management of autism.

### Case2

A 4-year-old boy with a history of autism was referred to one of the author’s (M.T.) private psychiatrist office for management of his symptoms. There was no reported comorbidity. Patient’s speech was fluent despite having difficulties in expressing himself. He previously received risperidone for the treatment of autism but he had a partial response to this medication. The patient was started on memantine 2.5 mg per day that was continued for 1 week and then increased to 5 mg per day. Similar to the first case, the child could only start to speak after a deep and audible breath. Otherwise, he was not able to talk. This problem was only noted at the beginning of speech and vanished after a while. Due to the fact that risperidone was discontinued 3 weeks prior to the initiation of memantine and the patient was not receiving any other medication, the new adverse effect was attributed to memantine. Since this difficulty was tolerable, memantine was continued at the same dose and was gradually increased to 7.5 mg per day. Interestingly, the speech difficulty was completely relieved despite continuation of memantine.

## Discussion

Memantine is an uncompetitive antagonist of NMDA receptor of glutamate. It has been used in several trials for the treatment of PDDs [18-21]. Interestingly, there are limited reports of stuttering development with glutamate antagonists such as lamotrigine and topiramate [8,22,23]. To the authors’ knowledge, there has been no previous report of stuttering induced or deteriorated by memantine use in children or adults. It should be noted that aphasia is one of the reported adverse effects of this medication [24]. However, this drug has been studied in the management of chronic post-stroke aphasia and showed improvement in the severity of this side effect [25]. Therefore, due to scant information on the causality of the relationship between memantine and aphasia, performing further studies in this area seem to be necessary to investigate this relationship.

The two patients in this report did not face any new stressful situations or conditions while under observation. Since both patients were only on memantine when their speech difficulties occurred, this drug was believed to be responsible for these adverse effects. Based on Naranjo adverse drug reaction probability scale, responsibility of memantine for occurrence of mentioned side effects was estimated as probable.

Although role of several neurotransmitters such as Gamma Amino Butyric Acid (GABA), serotonin and dopamine have been proposed in the pathogenesis of stuttering [2,26], it seems that the effects of dopamine are more prominent. It should be noted that the beneficial effects of anti-dopaminergic drugs such as risperidone, aripiprazole and olanzapine in the treatment of stuttering may confirm the role of dopamine in this disorder [2,27,28]. Additionally, due to the effects of memantine on glutamate receptors, this report may suggest a role for this neurotransmitter in the pathogenesis of some speech difficulties.

## Conclusion

The two cases presented in this report suggest that memantine may induce or exacerbate stuttering or cause difficulties in initiating speech in patients diagnosed with autism. The authors recommend that all clinicians should be aware of these bothersome speech-related side effects of memantine when deciding to prescribe this medication.

## Consent

Written informed consent was obtained from all the patients.

## Abbreviations

DSM-IV-TR: Diagnostic and statistical manual of mental disorders, fourth edition, text revision; GABA: Gamma amino butyric acid; ICD-10: International classification of diseases version 10; NMDA: N-methyl-d-aspartate; PDDs: Pervasive developmental disorders.

## Competing interests

The authors declared that they have no competing interest.

## Authors’ contributions

PG, JA, MT: interpreted the data, revised the manuscript critically for important intellectual content; and gave final approval of the version to be published. NN: contributed to acquisition of data, interpreted the data and involved and drafted the manuscript. All authors read and approved the final manuscript.
